# Bidirectional relationship between somatic pain and depression in Chinese adolescents: a cross-lagged study

**DOI:** 10.1186/s12889-026-28022-8

**Published:** 2026-06-18

**Authors:** Wang Xinzhu, Fu Kaixian, ZHang Yuxia, He Xinyu, Xu Gaicong

**Affiliations:** 1 Department of Education, Xichang university, Xichang City, China; 2 Division of Plan and Development, Xichang university, Xichang City, China; 3 Division of student affairs, Xichang university, Xichang City, China

**Keywords:** Bidirectional relationship, Somatic pain, Depression, Adolescent, Cross-lagged analysis

## Abstract

**Background:**

Somatic pain and depression are prevalent health concerns among Chinese adolescents. This study aims to examine the longitudinal reciprocal effects between these conditions in this population, explore the moderating role of gender, and propose recommendations to improve adolescent health.

**Materials and methods:**

Utilizing a cross-lagged design, this longitudinal study tracked a convenience sample of seventh- and eighth-grade students (aged 12–16) from two middle schools in Sichuan between April 2023 and April 2024. Measures included the Patient Health Questionnaire (PHQ-9) to assess depression and a five-item somatic pain scale. Analytical methods comprised descriptive statistics, Harman’s single-factor test, correlation analyses, t-tests, and structural equation modeling.

**Results:**

Cross-lagged analyses reveal a dynamic interplay between somatic pain and depression over time among Chinese adolescents, with gender serving as a significant moderator in this reciprocal relationship. Specifically, among adolescent boys, depression predicts somatic pain over a one-year interval, and somatic pain similarly predicts depression. In contrast, among adolescent girls, depression unidirectionally predicts somatic pain over the same period, with no significant effect observed in the reverse direction. Additionally, the association between somatic pain and depression is significantly stronger in adolescent girls than in boys at both time points. Furthermore, adolescent girls report higher levels of both somatic pain and depression compared to boys at both time points.

**Conclusion:**

Somatic pain and depression exhibit a dynamic interplay over time among Chinese adolescents, with gender significantly moderating their reciprocal relationship. These findings highlight the need for gender-specific intervention strategies and underscore the importance of addressing both somatic pain and depressive symptoms with equal priority in adolescent health management programs.

## Introduction

Adolescent mental and physical health has become a critical public health concern in contemporary Chinese society, attracting increasing attention from both academic circles and policymakers [1, 2]. Regarding physical health indicators, somatic pain is a prevalent issue among Chinese adolescents. A previous epidemiological study found that approximately one-third of primary school students regularly experience psychosomatic symptoms, such as recurrent headaches and abdominal pain, occurring at least once per week [3]. Another study involving 2,849 high school students reported prevalence rates of headache, abdominal pain, neck and shoulder pain, and low back pain at 30.3%, 20.9%, 32.8%, and 41.1%, respectively [4]. Regarding mental health indicators, depression has been identified as a prevalent psychological concern among adolescents in China. According to the China National Mental Health Development Report (2019–2020), the detection rate of depression among Chinese adolescents is 24.6%, with 7.4% classified as severe depression [5]. The Blue Book of National Depression 2022 reports that the prevalence of depression among adolescents ranges from 15% to 20% [6].

Meta-analyses indicate that depression and somatic pain commonly co-occur and are frequently comorbid, both in otherwise healthy individuals and in those diagnosed with depression [7–9]. In research, this comorbidity manifests in two primary ways. Firstly, there is a concurrent relationship, meaning that depressive symptoms and somatic pain are significantly correlated at any given time point. Numerous cross-sectional studies have established a close association between somatic pain and depression across populations of diverse age groups [10, 11]. Somatic pain is not only a manifestation of depression but is also closely related to the prognosis and treatment outcomes of depression [12]. Secondly, there exists a dynamic interplay between depression and somatic pain over time, whereby each can predict subsequent changes in the other. Research has revealed that depression may influence the longitudinal course of somatic pain [13, 14]. Follow-up studies have demonstrated that pain and depression can mutually affect each other over time [15–17]. For instance, a longitudinal study with a large sample of older adults revealed that somatic pain and depression can affect one another over a two-year period [16].

Emerging evidence suggests that somatic pain and depression may share common underlying mechanisms, including but not limited to: (1) neurobiological mechanisms, such as overlapping neural circuitry across multiple brain regions [18], dysregulation of monoamine neurotransmitter systems [19], and elevated inflammatory markers [20]; (2) psychological mechanisms, including the influence of depressive mood on increased pain sensitivity [21], catastrophizing cognitions about somatic symptoms that exacerbate depressive mood [22], and somatic pain contributing to heightened sensitivity to psychosocial stressors [21]. These mechanisms can be triggered by various shared factors that contribute to the comorbidity of depression and somatic symptoms, such as stress, family adversity, sleep disturbances, and unhealthy lifestyle habits [7, 23, 24].

The comorbid relationship between depression and somatic pain may be moderated by gender for three reasons. First, although inflammation is widely recognized as a core pathological mechanism underlying both depressive symptoms and chronic pain [25–27], a previous cross-lagged study demonstrated that the relationships among inflammation, depression, and pain differ by gender. Specifically, inflammation predicted increased depressive symptoms only in females, whereas somatic symptoms predicted elevated inflammation only in males [28]. Second, compared to males, a key characteristic of female endocrinology is its cyclical variation, which can lead to periodic fluctuations in mood states such as depression [29]. Third, Chinese cultural norms encourage males to exhibit strength and stoicism in the face of discomfort (e.g., pain) and to avoid expressing vulnerable emotions (e.g., crying), while granting females greater permission for emotional expression [30]. As a result, males are more likely to internalize stress [31]; thus, when boys experience unexplained somatic pain, they may be more prone to internalize it as negative emotion.

Currently, limited research has examined the longitudinal relationship between somatic pain and depression, as well as the moderating role of gender, in generally healthy adolescent populations. This gap hinders a deeper understanding of the reciprocal relationships and underlying mechanisms between depression and somatic pain across different gender groups, thereby impeding the development of targeted interventions for these conditions. Moreover, given the high prevalence of both depression and pain among Chinese adolescents, integrating these factors into a unified research framework not only enhances our understanding of adolescent depression but also facilitates the development of comprehensive intervention strategies. Therefore, this study aims to investigate how somatic pain and depression mutually influence each other over time in adolescents and how gender moderates their concurrent and longitudinal relationships. Based on the findings, we propose integrated intervention measures to improve the physical and mental health of Chinese adolescents.

Based on the comprehensive literature review presented above, this study proposes the following two research hypotheses:

- Hypothesis 1: Somatic pain and depression exert reciprocal influences on each other over time in Chinese adolescents.
- Hypothesis 2: Gender moderates the reciprocal relationship between somatic pain and depression in Chinese adolescents.

## Research methods

### Sampling procedure

Students from two middle schools in Sichuan Province were selected as participants in this study using a convenience sampling method. A longitudinal tracking design was employed, with two surveys conducted in April 2023 (Time 1) and April 2024 (Time 2). A total of 1,081 valid participants completed the survey at Time 1, while 966 valid participants completed the second survey, resulting in a loss of 115 students, corresponding to a loss rate of 10.64%. Participant attrition was primarily due to school transfers, leaves of absence, or extensive missing data. Questionnaires were excluded if they had two or more missing items on either the depression or somatic pain scales, or if any control variable (e.g., gender, age, ethnicity) was omitted. The results indicated no significant differences in gender (χ² = 0.35, *p* > 0.05), depression at Time1 (t = 1.07, *p* > 0.05), or somatic pain at Time 1(t = 1.34, *p* > 0.05) between participants who completed the survey at Time 1 and those lost at Time 2, suggesting that there was no systematic attrition.

### Instruments

The Patient Health Questionnaire (PHQ-9) was used to assess depression in adolescents [32]. This scale has been widely used in Chinese adolescent populations and has demonstrated good reliability and validity [33]. The scale comprises nine items, including “Feeling tired or having little energy.” Participants were asked to reflect on their emotional state over the past month and respond using a 4-point scale: 0 indicates “not at all”, 1 signifies “several days”, 2 represents “more than half the days”, and 3 stands for “nearly every day”. The Cronbach’s alpha coefficients for the first wave-survey and the second wave-survey were 0.89 and 0.91, respectively. In this study, a higher mean score on the scale corresponds to more severe depression.

The five items used to measure somatic pain in this study were adapted from two previous studies [34, 35]. These items assess somatic pain in five locations: headache, stomach pain, neck and shoulder pain, back pain, and pain in the arms and legs. Participants were asked to recall any somatic pain they had experienced in the past month, excluding pain caused by menstruation, sports injuries, accidental impacts, or clinically evident illnesses(e.g., pain from conditions such as gastric disorders or arthritis, as diagnosed by a physician). They were instructed to respond using a five-point scale: “0” indicated “never,” “1” indicated “occasionally,” “2” indicated “sometimes,” “3” indicated “often,” and “4” indicated “always.” One of the items is: “During the past month, I had headaches.” The somatic pain index is computed by averaging the scores of the five items, with higher mean scores indicating greater frequency of somatic pain. The Cronbach’s alpha reliability coefficients for the first and second wave surveys were 0.76 and 0.78, respectively.

### Ethic statements

This investigation was conducted in strict compliance with all relevant guidelines and regulations and received approval from the Academic Committee of Xichang University (Approval No. 2023031). The first wave of the survey took place on the morning of April 5, 2023, and the second wave on the morning of April 10, 2024. During the three months preceding the first wave, we obtained written informed consent from all student guardians and securely stored these documents in the archival cabinets of the Teacher Education College.

### Data processing method

The study first employed Harman’s single-factor test in SPSS 27.0 to assess common method bias, followed by descriptive statistics and Pearson correlation analyses to preliminarily validate the concurrent comorbidity between depression and somatic pain. Subsequently, a structural equation model based on the entire sample was constructed using Mplus 8.1 to examine the cross-temporal comorbidity between depression and somatic pain. Finally, separate structural equation models were established for adolescent boys and girls, with the Wald chi-square test applied to verify how gender moderates the comorbid relationship between depression and somatic pain. To control for the effects of non-normal data distribution, extreme values, and potential Type I error, this study employed the MLR estimator when establishing the structural equation model in Mplus and reported 95% confidence intervals.

## Research results

### Basic demographics of the valid sample

The final sample for this study consisted of 966 students who completed all assessments. Their basic characteristics are presented in Table 1.

**Table 1:** Basic characteristics of the valid samples(*N* = 966)

Variable	Levels	Frequency	Percentage
Age (M = 13.78, SD = 0.81)	12	11	1.1
	13	386	40
	14	402	41.6
	15	146	15.1
	16	21	2.2
Gender	Boy	474	49.1
	Girl	492	50.9
Grade	7	225	23.3
	8	741	76.7
Family residence	Village	209	21.6
	Town	322	33.3
	City	435	45.0
Ethnicity	*Han*	784	81.2
	Minority	182	18.8
Height (M = 160.38, SD = 8.12)	Boy	M = 163.41	SD = 8.85
	Girl	M = 157.47	SD = 6.05

*M *mean, *SD *Standard deviation. Age, grade, and height were collected during the second wave of the survey

### Common method deviation test

The Harman single-factor test revealed two factors with eigenvalues greater than 1 when analyzing the relationship between depression and somatic pain measurements in both surveys. The first factor accounted for 43.05% and 46.61% of the variance, respectively, both below the 50% threshold. Therefore, this study did not demonstrate significant common method bias [36].

### Descriptive statistics and correlation analysis

The mean, standard deviation, and correlation coefficients of the major variables for the entire sample are presented in Table 2.

**Table 2:** Descriptive data and pearson correlations in the whole sample(*N* = 966)

	M	SD	DepressionT1	DepressionT2	Somatic pain T1	Somatic pain T2
Depression T1	1.14	0.77	1			
Depression T2	1.12	0.82	0.63^**^	1		
Somatic pain T1	0.71	0.70	0.57^**^	0.42^**^	1	
Somatic pain T2	0.73	0.73	0.48^**^	0.61^**^	0.60^**^	1

T1 = at Time 1, T2 = at Time 2. ** *p* < 0.01, * *p* < 0.05

Table 2 demonstrates a positive association between depression and somatic pain across two time points, with correlation coefficients (r) ranging from 0.42 to 0.48 (all *p* < 0.01). Additionally, there is a significant positive correlation between depression and somatic pain at each individual time point, with *r* = 0.57 at T1 and *r* = 0.61 at T2 (both *p* < 0.01).

According to a previous study by Schober and Boer(2018), a correlation coefficient ranging from 0.40 to 0.69 indicates a moderate correlation [37]. Therefore, in the adolescent population, depression and somatic pain are moderately and positively correlated both concurrently and over time.

Descriptive data and Pearson correlations among the variables for adolescent boys and girls are presented in Table 3. Additionally, independent samples t-tests were conducted to examine gender differences in both depressive symptoms and somatic pain; the detailed results are also presented in Table 2.

**Table 3:** Descriptive data and pearson correlations in different genders

	Boys		Girls		t	Depression T1	Depression T2	Somatic pain T1	Somatic pain T2
	M	SD	M	SD					
Depression T1	0.98	0.72	1.30	0.79	-6.03^**^	1	0.62^**^	0.58^**^	0.47^**^
Depression T2	0.92	0.76	1.32	0.84	-7.38^**^	0.60^**^	1	0.38^**^	0.60^**^
Somatic pain T1	0.55	0.62	0.87	0.73	-7.32^**^	0.52^**^	0.41^**^	1	0.60^**^
Somatic pain T2	0.51	0.62	0.95	0.75	-9.70^**^	0.42^**^	0.55^**^	0.51^**^	1

The lower left matrix is for boys, and the upper right matrix is for girls. ^**^p<0.01, ^*^p<0.05. T1 = at Time 1, T2 = at Time 2

Table 3 shows that the correlations between depression and somatic pain closely mirror those observed in the entire sample at the corresponding time points (see Table 2).

In addition, Table 3 shows that in both waves of the survey, adolescent girls reported higher levels of somatic pain and depression than adolescent boys.

### The cross-lagged panel analyses

#### The reciprocal relationship between somatic pain and depression

**Fig. 1:**
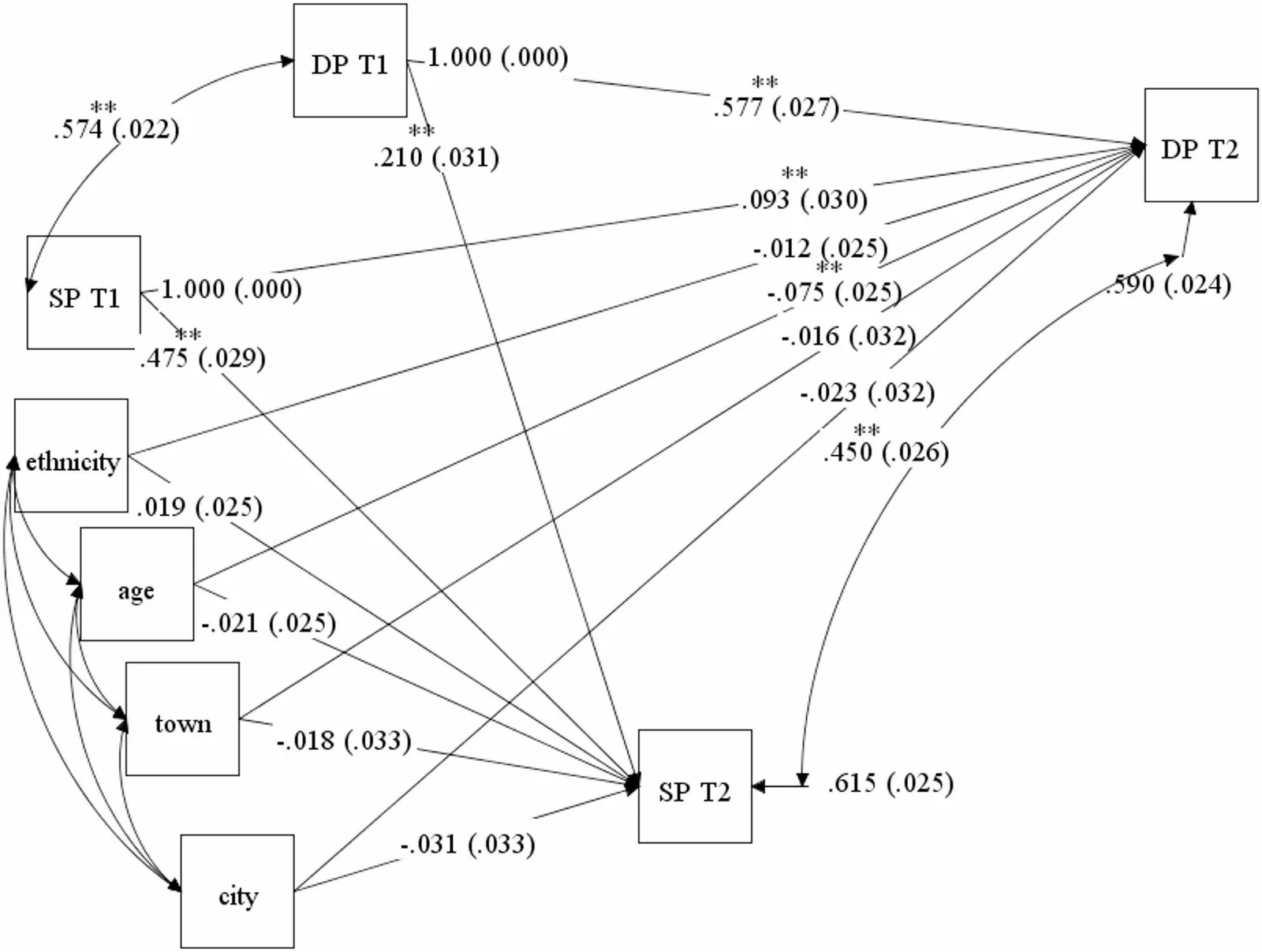
The results of cross-lagged analysis on the entire sample. Note: All path coefficients were based on stdyx. ** *p*<0.01, * *p*<0.05. DP=depression, SP=somatic pain; T1= at Time 1, T2=at Time 2. Reference groups for the categorical variables were male (for gender), village (for family residence), and Han (for ethnicity)

This study employed cross-lagged analysis to test the research hypothesis. Gender, age, ethnicity, and family residence were controlled for. First, a cross-lagged analysis of the saturated model was conducted using the entire sample, and the results are presented in Fig. 1.

As illustrated in Fig. 1, the analysis of the entire sample revealed that depression at Time 1 significantly predicted somatic pain at Time 2 (β = 0.210, SE = 0.031, 95% CI [0.150, 0.270], *p* < 0.01), while somatic pain at Time 1 also significantly predicted depression at Time 2 (β = 0.093, SE = 0.030, 95% CI [0.034, 0.152], *p* < 0.01).

Additionally, age had a significant effect on depression at Time 2(β = -0.075, SE = 0.025, 95% CI [-0.123, -0.026], *p* < 0.01).

**Fig. 2:**
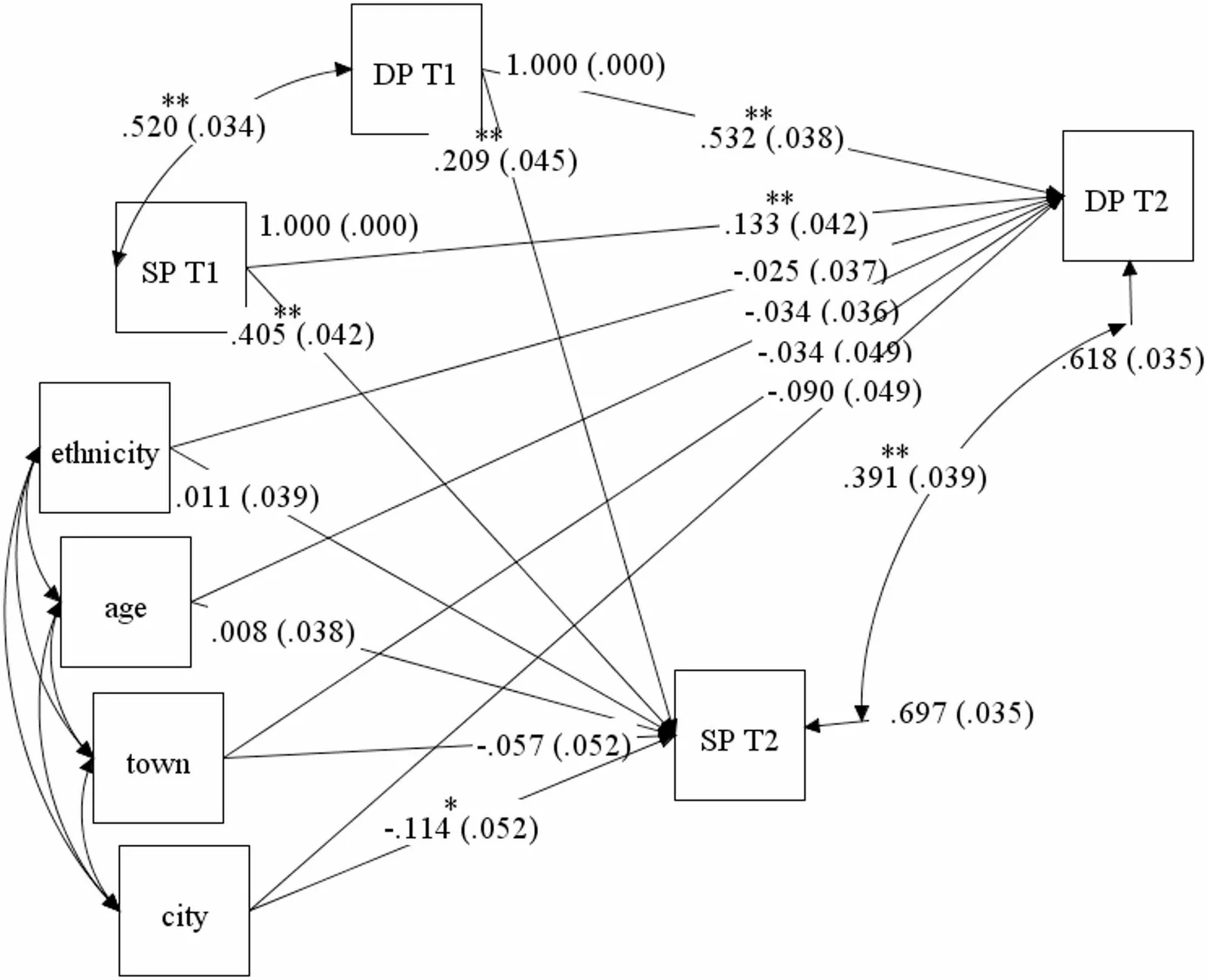
The results of cross-lagged analysis among adolescent boys. Note: All path coefficients were based on stdyx. ** *p*<0.01, * *p*<0.05. DP=depression, SP=somatic pain; T1= at Time 1, T2=at Time 2. Reference groups for the categorical variables were male (for gender), village (for family residence), and Han (for ethnicity)

Second, the present study conducts a cross-lagged analysis among adolescent boys. The results are displayed in Fig. 2.

Figure 2 demonstrated that depression at Time 1 had a significant effect on somatic pain at Time 2 (β = 0.209, SE = 0.045, 95%CI [0.322, 0.488], *p* < 0.01), and somatic pain at Time 1 had a significant effect on depression at Time 2 (β = 0.133, SE = 0.042, 95%CI[0.051, 0.216], *p* < 0.01).

Furthermore, family residence had a significant effect on somatic pain at T2 (β = -0.114, SE = 0.052, 95% CI [-0.215, -0.013], *p* < 0.05). Compared to students from villages, those from cities reported significantly lower levels of somatic pain.

**Fig. 3:**
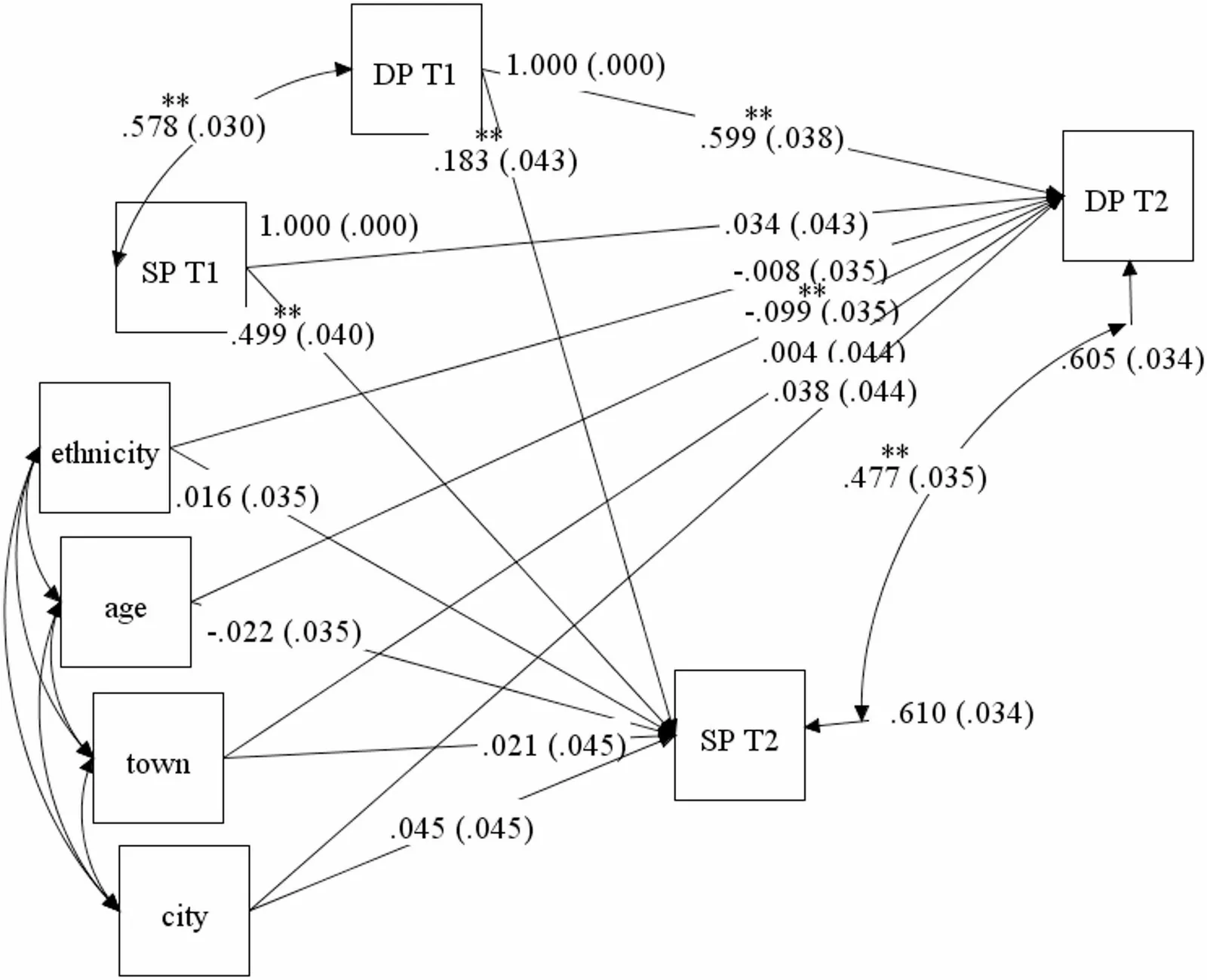
The results of cross-lagged analysis among adolescent girls. Note: All path coefficients were based on stdyx. ** *p*<0.01, * *p*<0.05. DP=depression, SP=somatic pain; T1= at Time 1, T2=at Time 2. Reference groups for the categorical variables were male (for gender), village (for family residence), and Han (for ethnicity)

Lastly, the present study conducted a cross-lagged analysis among adolescent girls. The results are shown in Fig. 3.

Figure 3 demonstrated that depression at Time 1 had a significant effect on somatic pain at Time 2 (β = 0.183,SE = 0.043, 95%CI[0.098, 0.267], *p* < 0.01), while somatic pain at Time 1 had no significant effect on depression at Time 2 (β = 0.034, SE = 0.043, 95%CI[-0.051, 0.119], *p* > 0.05).

Furthermore, age demonstrated a significant negative effect on depression (β = -0.099, SE = 0.035, 95% CI [-0.168, -0.030], *p* < 0.01).

Therefore, Hypothesis 1, which posits that somatic pain and depression exert reciprocal influences on each other over time in Chinese adolescents, was strongly supported in the overall sample and among adolescent boys. However, this hypothesis was not supported among adolescent girls, as the relationship was unidirectional: depression significantly influenced somatic pain, but the reverse effect was not significant.

#### The moderation role of gender in the reciprocal relationship between somatic pain and depression

We used Wald test of parameter constraints to examine the moderation role of gender in the reciprocal relationship between somatic pain and depression. The model demonstrated excellent fit in boy subsample: χ²(8) = 9.365, CFI = 0.997, TLI = 0.995, RMSEA = 0.019 (90% CI: 0.000, 0.059), SRMR = 0.029. Furthermore, the Wald test revealed that the magnitude of the cross-lagged path from depression at Time 1 to somatic pain at Time 2 (β = 0.209, *p* < 0.01) was not statistically different from the reverse path from somatic pain at Time 1 to depression at Time 2 (β = 0.133, *p* < 0.01; Δχ²(1) = 0.057, *p* > 0.05; see Fig. 2). In girl subgroup the model also demonstrated excellent fit: χ²(8) = 5.396, CFI = 1.000, TLI = 1.000, RMSEA = 0.000 (90% CI: 0.000, 0.040), SRMR = 0.018. The Wald test further confirmed that the effect of depression at Time 1 on somatic pain at Time 2 (β = 0.183, *p* < 0.01) was significantly stronger than the reverse effect of somatic pain at Time 1 on depression at Time 2 (β = 0.034, *p* > 0.05; Δχ²(1) = 3.89, *p* < 0.05; see Fig. 3).

In summary, among adolescent boys, somatic pain and depression influence each other over time in a symmetrical manner. However, among adolescent girls, depression predicts somatic pain, with no significant reverse effect. Therefore, Hypothesis 2, which proposes that gender moderates the reciprocal relationship between somatic pain and depression in Chinese adolescents, is strongly supported.

#### Gender differences in the autocorrelation between somatic pain and depression

The present study used the Wald Test of parameter constraints to compare the autocorrelation between somatic pain and depression across different genders. The magnitude of the autocorrelation between depression at Time 1 and somatic pain at Time 1 among adolescent girls (*r* = 0.578; see Fig. 3) is significantly greater than that among their male counterparts (*r* = 0.520; see Fig. 2; Δχ² = 7.090, df = 1, *p* < 0.01). Similarly, the magnitude of the autocorrelation between depression at Time 2 and somatic pain at Time 2 among adolescent girls (*r* = 0.477; see Fig. 3) is significantly greater than that among their male counterparts (*r* = 0.391; see Fig. 2; Δχ² = 6.336, df = 1, *p* < 0.01). Consequently, somatic pain demonstrates a stronger association with depression among adolescent boys than among their male counterparts at the cross-sectional level.

#### Effects of control variables

The current study identified a significant negative effect of age on depression at Time 2 (see Figs. 1 and 3), indicating that older adolescents reported lower levels of depressive symptoms. This pattern may be explained by their greater psychological maturity and consequently superior emotion regulation capabilities [38], which enables them to select more effective strategies to cope with stress, thereby reducing depressive symptoms [39].

Furthermore, among adolescent boys, family residence had a significant negative effect on somatic pain at Time 2. This indicates that boys from urban areas reported fewer somatic pain symptoms compared to their rural counterparts. A plausible explanation for this finding is the generally greater availability of healthcare resources and health-related infrastructure in urban settings, which may contribute to better physical health outcomes for adolescents [40].

## Discussion

### Levels of somatic pain and depression and in adolescents

This study reveals that the mean scores for somatic pain were 0.71 and 0.73 at the two time points, respectively, measured on a 0–4 Likert scale. These results indicate that adolescents, on average, experienced somatic pain with a frequency approaching “occasional.” This finding is consistent with earlier observations reported by Hesketh et al. [3].

Meanwhile, the mean scores of depressive symptoms among the sampled adolescents were 1.14 and 1.12, respectively, across the two survey waves, as measured on a 0–3 Likert scale. This suggests that, on average, adolescents reported depressive symptoms approaching moderate levels—a finding consistent with previous epidemiological studies [5].

Furthermore, gender significantly influenced both somatic pain and depression, with adolescent girls reporting more frequent pain symptoms and higher levels of depression than their male counterparts (see Table 3). This observed gender disparity can be attributed to two primary factors. First, within the Chinese educational system, adolescent girls often experience greater academic pressure and tend to invest more effort in their studies, which may contribute to increased stress-related symptoms [31]. Second, prevailing cultural norms in China permit females greater freedom to express emotional and physical distress, whereas males are socialized to demonstrate stoicism and endurance in the face of discomfort [30]. Consequently, females may be more inclined to openly report symptoms of depression and somatic pain.

### The reciprocal relationship between somatic pain and depression

This study employed structural equation modeling to analyze longitudinal survey data, aiming to examine the reciprocal relationships between somatic pain and depression over time among Chinese adolescents. We initially constructed a saturated structural equation model based on the overall sample, which revealed that somatic pain and depression mutually influence each other over time in adolescents (see Fig. 1). This finding supports Hypothesis 1, which posits that somatic pain and depression affect each other in Chinese adolescents. This result aligns with previous longitudinal studies [15–17]. For example, a study involving newly diagnosed adults with arthritis demonstrated that bodily pain and depression influence one another over time [15]. Additionally, another study tracking older adults found that pain at baseline independently predicted the development of depression two years later, while baseline depression predicted the experience of pain two years later [16].

However, when the data for boys and girls were analyzed separately, the results diverged. Among boys, Hypothesis 1 remained supported, indicating that somatic pain and depression exhibit a bidirectional interaction over a one-year interval (see Fig. 2). In contrast, among girls, baseline depression significantly influenced somatic pain one year later, whereas baseline somatic pain did not significantly predict the level of depression after one year (see Fig. 3). Consequently, Hypothesis 1 was not supported among adolescent girls.

The results of the Wald test of parameter constraints indicate gender differences in the bidirectional relationships between somatic pain and depression over time among adolescents. Specifically, for adolescent boys, the test revealed that the effect of somatic pain at Time 1 on depression at Time 2 is not significantly different from the effect of depression at Time 1 on somatic pain at Time 2 (see Fig. 2). This suggests a symmetrical relationship between somatic pain and depression in males, where both constructs influence each other to a similar extent over time. In contrast, for girls, the predictive relationship was asymmetrical: the path from depression to subsequent somatic pain was both statistically significant and substantially larger in magnitude compared to the non-significant path from somatic pain to subsequent depression (see Fig. 3). Therefore, Hypothesis 2, which posits that gender moderates the reciprocal relationship between somatic pain and depression in Chinese adolescents, is strongly supported.

In contrast to boys, the effect of depression on subsequent somatic pain is stronger than the reverse effect among girls. Several mechanisms may explain this pattern. During puberty, adolescent boys experience a relatively stable increase in testosterone levels [41], which may synchronize the development of negative emotions and somatic symptoms [42]. Additionally, within Chinese cultural norms, male adolescents are expected to demonstrate strength and stoicism by enduring discomfort without overt expression [43]. Consequently, when experiencing somatic pain, boys may be more likely to internalize these sensations as negative emotions such as depression, anxiety, or anger. This internalization process explains why somatic pain at Time 1 predicts increased depressive symptoms among boys. Although girls experience cyclical hormonal fluctuations that can simultaneously affect both mood and somatic symptoms [44], they generally receive greater social permission for emotional expression. This allowance may enable them to alleviate somatic discomfort through verbal and emotional outlets, preventing the internalization of somatic pain at Time 1 into subsequent depressive mood.

Furthermore, the Wald Test of parameter constraints revealed that the strength of the association between somatic pain and depression at both time points was significantly greater among adolescent girls compared to their male counterparts. This finding indicates a higher likelihood of co-occurring somatic pain and depression among adolescent girls, a correlation that has not been explicitly emphasized in prior studies [10, 19]. This pattern can be primarily explained by two factors. First, adolescent girls generally have significantly more insomnia symptoms than boys [45]. Insomnia, in turn, contributes to both depressed mood and somatic pain [46], with its influence on pain being 3.5 times stronger in girls than in boys [47]. Second, Traditional Chinese culture, influenced by Confucianism and patriarchy, still has a negative impact on female mental well-being [48], and cultural norms emphasizing emotional restraint and collectivist values may encourage females to express distress through physical symptoms rather than psychological complaints [49, 50]. The confluence of these biological and sociocultural mechanisms leads to a stronger cross-sectional association between depression and somatic pain measurements at any given time point among adolescent girls.

### Research implications

This longitudinal study examined the bidirectional relationships between somatic pain and depression in adolescents. Cross-lagged analyses revealed that somatic pain and depression mutually influence each other over time, with gender moderating these associations. Specifically, a symmetrical relationship was observed in boys, where somatic pain and depression exerted comparable reciprocal effects over time. In contrast, an asymmetrical relationship was found in girls, with depression showing a stronger predictive effect on subsequent somatic pain than the reverse. These findings highlight the critical importance of incorporating gender-specific perspectives when investigating the dynamic interplay between somatic symptoms and mental health outcomes across developmental stages.

This study presents three significant practical implications for adolescent health management within the Chinese context. First, both families and educational institutions should prioritize the identification and intervention of somatic pain among adolescents. Given the high prevalence of somatic symptoms in this population, establishing effective pain management protocols is essential to promote physical well-being, enhance quality of life, and optimize academic performance [51, 52].

Second, the development of depression interventions should incorporate comprehensive somatic pain management strategies. Empirical evidence indicates a robust bidirectional relationship between depression and somatic pain, with particularly pronounced co-occurrence and mutual influence observed in male adolescents. This finding is supported by a recent study demonstrating that effective management of comorbid pain in primary depressive disorders significantly contributes to the alleviation of depressive symptoms [53]. To optimize intervention outcomes, it is recommended to establish an integrated school-medical collaborative framework that synergistically combines psychological counseling services with evidence-based pain management protocols, thereby addressing the psychosomatic complexity of adolescent depression.

Third, adolescent girls require increased allocation of resources for targeted health management interventions. The present study revealed that adolescent girls exhibit a higher prevalence of somatic pain and elevated levels of depressive symptoms compared to adolescent boys. Furthermore, the association between depression and somatic pain was found to be more pronounced at two time points in adolescent girls than in boys. These findings underscore the necessity for educational institutions and healthcare providers to implement gender-specific health management strategies, with particular emphasis on addressing the unique mental and physical health needs of adolescent girls.

### Advantages and limitations

This study offers two significant advantages. First, it employs longitudinal data to examine the relationship between somatic pain and depression among Chinese adolescents, thereby addressing a research gap and enhancing the understanding of families and educators regarding the interplay between somatic pain and depression in this population. Second, the study reveals that somatic pain and depression interact in distinct patterns between boys and girls, a finding not previously identified in similar research [54, 55]. This suggests that gender-specific strategies should be implemented in interventions targeting depression and somatic pain.

Several methodological limitations should be acknowledged when interpreting our findings. First, the one-year interval between data collection waves may not adequately capture the long-term developmental trajectories and reciprocal dynamics between somatic pain and depression during adolescence. Second, the reliance on convenience sampling from only two middle schools in Sichuan Province limits the generalizability of our results to the broader adolescent population across China’s diverse geographical and socioeconomic contexts. Third, the measurement of somatic pain, restricted to five specific locations using a brief scale, may not fully capture the spectrum of somatic symptoms experienced by adolescents, potentially compromising the construct validity of our pain assessment. Fourth, although we identified gender as a significant moderator, the underlying mechanisms explaining these gender differences in the reciprocal relationship between somatic pain and depression remain unclear, representing an important gap in our understanding of these psychosomatic interactions. Finally, the lack of strict correction for multiple comparisons increases the risk of Type I errors (i.e., false-positive findings [56]. Although confidence intervals were provided, this limitation constrains the internal validity of the present study and necessitates replication in future independent samples. Future research should address these limitations through longitudinal designs with extended follow-up periods, more representative samples, comprehensive multidimensional pain assessments, and theoretically driven investigations of gender-specific mechanisms. Such methodological advancements would significantly enhance our understanding of the complex interplay between somatic symptoms and mental health outcomes during this critical developmental period.

## Conclusion

This study employed a two-wave longitudinal design to examine the reciprocal relationships between somatic pain and depression among Chinese adolescents. Cross-lagged panel analysis revealed significant bidirectional associations between somatic pain and depression over a one-year interval, with gender serving as a significant moderator of these relationships. Specifically, a symmetrical reciprocal relationship was observed in adolescent boys, wherein somatic pain and depression exerted comparable mutual influences over time. In contrast, adolescent girls exhibited an asymmetrical pattern, characterized by a significant and stronger predictive effect of depression on somatic pain, while the reverse pathway was non-significant. Furthermore, the cross-sectional associations between somatic pain and depression were consistently stronger among adolescent girls at both time points compared to boys. Finally, adolescent girls consistently reported higher levels of both somatic pain and depression than their male counterparts. These findings enhance our understanding of the relationship between somatic pain and depression among adolescents and can inform effective strategies to improve adolescent health in China.

## Data Availability

The raw data for this study is available at [https://www.scidb.cn/en/anonymous/TnpBSmYy].
